# Comparative genetics of a highly divergent *DRB* microsatellite in different macaque species

**DOI:** 10.1007/s00251-008-0333-z

**Published:** 2008-10-28

**Authors:** Nanine de Groot, Gaby G. M. Doxiadis, Annemiek J. M. de Vos-Rouweler, Natasja G. de Groot, Ernst J. Verschoor, Ronald E. Bontrop

**Affiliations:** Department of Comparative Genetics and Refinement, Biomedical Primate Research Centre, P.O. Box 3306, 2280 GJ Rijswijk, The Netherlands; Department of Comparative Genetics and Refinement, Biomedical Primate Research Centre, P.O. Box 3306, 2280 GJ Rijswijk, The Netherlands; Department of Comparative Genetics and Refinement, Biomedical Primate Research Centre, P.O. Box 3306, 2280 GJ Rijswijk, The Netherlands; Department of Comparative Genetics and Refinement, Biomedical Primate Research Centre, P.O. Box 3306, 2280 GJ Rijswijk, The Netherlands; Department of Virology, Biomedical Primate Research Centre, Lange Kleiweg 139, P.O. Box 3306, 2280 GJ Rijswijk, The Netherlands; Department of Comparative Genetics and Refinement, Biomedical Primate Research Centre, P.O. Box 3306, 2280 GJ Rijswijk, The Netherlands

**Keywords:** MHC, Nonhuman primates, Evolution, Microsatellites, Macaques

## Abstract

The *DRB* region of the major histocompatibility complex (MHC) of cynomolgus and rhesus macaques is highly plastic, and extensive copy number variation together with allelic polymorphism makes it a challenging enterprise to design a typing protocol. All intact *DRB* genes in cynomolgus monkeys (*Mafa*) appear to possess a compound microsatellite, DRB-STR, in intron 2, which displays extensive length polymorphism. Therefore, this STR was studied in a large panel of animals, comprising pedigreed families as well. Sequencing analysis resulted in the detection of 60 *Mafa-DRB* exon 2 sequences that were unambiguously linked to the corresponding microsatellite. Its length is often allele specific and follows Mendelian segregation. In cynomolgus and rhesus macaques, the nucleotide composition of the DRB-STR is in concordance with the phylogeny of exon 2 sequences. As in humans and rhesus monkeys, this protocol detects specific combinations of different DRB-STR lengths that are unique for each haplotype. In the present panel, 22 *Mafa-DRB* region configurations could be defined, which exceeds the number detected in a comparable cohort of Indian rhesus macaques. The results suggest that, in cynomolgus monkeys, even more frequently than in rhesus macaques, new haplotypes are generated by recombination-like events. Although both macaque species are known to share several identical *DRB* exon 2 sequences, the lengths of the corresponding microsatellites often differ. Thus, this method allows not only fast and accurate *DRB* haplotyping but may also permit discrimination between highly related macaque species.

## Introduction

The *Mhc-DRB* region in various primate species displays abundant levels of allelic variation (polymorphism) and diversity (gene copy number variation). However, the balance between these two phenomena can differ significantly, depending on the species studied (Brändle et al. 1992; Grahovac et al. 1992; Mayer et al. 1992; Schönbach et al. 1993; Gongora et al. 1997; Antunes et al. 1998; Doxiadis et al. 2000; de Groot et al. 2008). In humans, nine different genes have been characterized, designated *HLA-DRB1–9* (Marsh et al. 2005), and equivalents have been detected in various nonhuman primate species (Kasahara et al. 1990; Klein et al. 1991; Brändle et al. 1992; Kenter et al. 1992; Mayer et al. 1992; Trtkova et al. 1993; Slierendregt et al. 1994; Bontrop et al. 1995; Leuchte et al. 2004; Doxiadis et al. 2006a). A region configuration is defined by the unique combination of distinct *DRB* genes present per haplotype. In humans, five major *DRB* region configurations are known (DR1, DR8, DR51, DR52, DR53), whereas in chimpanzees at least six and in rhesus macaques more than 30 different region configurations have been defined (Gyllensten et al. 1991; Mayer et al. 1992; Slierendregt et al. 1994; Gongora et al. 1997; Khazand et al. 1999; Doxiadis et al. 2000; Bontrop 2006; O'Connor et al. 2007; de Groot et al. 2008). In contrast, a New World primate species like the common marmoset (*Callithrix jacchus*) lacks region configuration polymorphism (Antunes et al. 1998; Doxiadis et al. 2006b).

Cynomolgus monkeys (*Macaca fascicularis*) are frequently used as animal models for immune-related diseases or in transplantation studies (Bosinger et al. 2004; Jonker et al. 2004; Langermans et al. 2005; Mueller et al. 2005; Wiseman et al. 2007; Wojcechowskyj et al. 2007). Resistance or susceptibility to certain immune-related diseases as for example HIV/SIV appears to be correlated to the geographic origin of rhesus as well as cynomolgus macaques (Wiseman et al. 2007; Goulder and Watkins 2008). Additionally, several studies have proven that cynomolgus macaques from different origins also vary concerning the genetic diversity of their mtDNA, Y chromosome, and autosomal markers with Mauritius animals being the most homogenous due to a founder effect (Smith et al. 2007; Tosi and Coke 2007; Blancher et al. 2008; Bonhomme et al. 2008). To get a first idea of the origin and the diversity of the cynomolgus monkeys selected for this study, mtDNA variation was analyzed as this has been proven to be a suitable method (Tosi et al. 2003; Smith and McDonough 2005; Kyes et al. 2006; Smith et al. 2007).

Although the *Mafa-DR* region is not as thoroughly analyzed as for the rhesus monkey (*Macaca mulatta*; *Mamu-DR*), recent studies have indicated that the levels of diversity at these regions are at least comparable (Blancher et al. 2006; Doxiadis et al. 2006a; O'Connor et al. 2007). Because of the complexity of the region, until now, laborious methods like cloning, followed by sequencing of the most polymorphic exon 2 or full-length *DRB*, are the only means available for accurate typing. It is clear that more straightforward typing protocols are needed that can easily be implemented by other laboratories.

A complex repeat, designated D6S2878 or DRB-STR, maps in close proximity to exon 2 and is present in the majority of all *HLA*- and *Mamu*-*DRB* genes (Andersson et al. 1987; Epplen et al. 1997; Bergstrom et al. 1999; Doxiadis et al. 2007). This entity is located at the beginning of intron 2 and has a compound character (Riess et al. 1990; Trtkova et al. 1995; Bergstrom et al. 1999; Kriener et al. 2000; Doxiadis et al. 2007). Genotyping of a large panel of cells, covering most of the known *HLA*- and *Mamu-DRB* specificities, resulted in the definition of unique DRB-STR patterns that appeared to be characteristic for a certain haplotype (Doxiadis et al. 2007). Therefore, this microsatellite may represent a promising marker for the *DRB* haplotyping of other macaque species. Consequently, a representative panel including several families was chosen to investigate whether it is possible to set up a high-resolution *DRB* haplotyping protocol for cynomolgus monkeys.

## Materials and methods

### Samples and genomic DNA isolation

All of the 71 cynomolgus macaques but two unrelated males (Cyn81 and Cyn83) used for studying *Mafa-DRB* belong to an outbred colony that is housed at the University of Utrecht, The Netherlands, and are members of four different pedigreed families (Juanita, Rastafa, Sayonara, and Alfa). For mtDNA analysis, representatives of these four families have been analyzed, and members of other cynomolgus families (Epha, Harpo, Bilboa, Cleo, Hoeba, and Cornea) and some unrelated monkeys (Cyn80, Vip, Clint, K66, and K95) have been included. Genomic DNA was extracted from EDTA blood samples or from immortalized B-cell lines using a standard salting out procedure.

### mtDNA analysis

DNA was obtained as described above, or was extracted from feces in 96% ethanol using the QIAamp DNA stool mini kit (Qiagen, GmbH, Germany) according to the manufacturer's recommendations. In particular, the 3′ part of the 12S rRNA gene is helpful in distinguishing species and origin-related variability (van der Kuyl et al. 1995, 2000; Doxiadis et al. 2003; Tosi et al. 2003). Amplification of part of the mitochondrial 12S rRNA gene, purification, and sequencing was performed essentially according to published methods (Kocher et al. 1989; Doxiadis et al. 2003). The data were analyzed using the Sequence Navigator program (Applied Biosystems). Unreported sequences resulting from at least two independent PCR reactions have been deposited in the EMBL/GeneBank database (accession numbers FM179743–FM179751).

### Phylogenetic analysis of mtDNA

Multiple sequence alignments of the 12S rRNA sequences were obtained of newly generated mtDNA sequences (accession numbers see above) together with published mtDNA sequences of animals of documented origin (Doxiadis et al. 2003; Tosi et al. 2003) using MacVector v9.5.2 (MacVector, Inc., Cambridge, UK). Mr Bayes v3.1.2 was used for Bayesian interference of the phylogenetic relationships of the published (Tosi et al. 2003; Doxiadis et al. 2006a) and newly detected mtDNA sequences (373 bp). Four Monte Carlo Markov chains (MCMCs) were run simultaneously for 1 × 10^6^ generations, with posterior sampling of the trees after every 100 generations and a burn-in after 1,250 generations (Huelsenbeck and Ronquist 2001; Ronquist and Huelsenbeck 2003).

### STR-DRB genotyping

Amplification of the relevant DNA segment in cynomolgus macaques was performed as described for rhesus macaques using the same primer sets (Doxiadis et al. 2007). Briefly, the relevant DNA segment in macaques was amplified with a forward primer located at the end of exon 2 (*5′ Mamu-DRB*-STR—TTC ACA GTG CAG CGG CGA GGT) and two labeled reverse primers in intron 2 (3′ *Mamu-DRB*-STR_VIC—ACA CCT GTG CCC TCA GAA CT and 3′ *Mamu-DRB*-STR_FAM_1007—ACA TCT GTG TCC TCA GAC CT). The labeled primers were synthesized by Applied Biosystems (Foster City, USA) and the unlabelled primers by Invitrogen (Paisley, Scotland). The PCR reaction was performed in a 25-μl reaction volume containing 1 U of *Taq* polymerase (Invitrogen) with 0.6 μM of the unlabeled forward primer, 0.4 μM of the VIC labeled reversed primer, 0.2 μM of the FAM-labeled reversed primer, 2.5 mM MgCl_2_, 0.2 mM of each dNTP, 1 × PCR buffer II (Invitrogen), and 100 ng DNA. The cycling parameters were a 5-min 94°C initial denaturation step, followed by five cycles of 1 min at 94°C, 45 s at 58°C, and 45 s at 72°C. Then the program was followed by 25 cycles of 45 s at 94°C, 30 s at 58°C, and 45 s at 72°C. A final extension step was performed at 72°C for 30 min. The amplified DNA was prepared for genotyping according to the manufacturer's guidelines and analyzed on an ABI 3130 genetic analyzer (Applied Biosystems). STR analysis was performed with the Genemapper program (Applied Biosystems) and all samples were analyzed at least twice.

### PCR, cloning, and sequencing of *Mafa*-*DRB*

Sixty different *Mafa-DRB* alleles were sequenced from exon 2 to intron 2, including the microsatellite. Therefore, we used the same primers and PCR reaction as described for rhesus macaques (Doxiadis et al. 2007). The 18 unreported *Mafa*-*DRB* sequences have been deposited in the EMBL/GeneBank database (accession numbers AM910925–AM910929, AM911049, AM911051, AM911053–AM911060, AM911062, AM911063, and AM911065) and are also available via the IPD/MHC database (www.ebi.ac.uk/ipd/mhc/nhp; European Bioinformatics Institute, Cambridge, UK). Alleles have been named according to a standardized nomenclature protocol (Klein et al. 1990; Robinson et al. 2003; Ellis et al. 2006).

### Phylogenetic analysis of *DRB* exon 2 sequences

Multiple sequence alignments of exon 2 of *Mafa*-*DRB* sequences generated in this study together with published *Mafa*- and *Mamu*-*DRB* sequences from the NCBI database were created using MacVector™ version 8.1.1 (Oxford Molecular Group), followed by a phylogenetic analysis performed using PAUP version 4.0b.10 (Swofford 2002). Pairwise distances were calculated using the Kimura two-parameter model for creating a phylogram. Confidence estimates of grouping were calculated according to the bootstrap method generated from 1,000 replicates.

## Results and discussion

### Origin of cynomolgus macaques, defined by mtDNA analysis

**Fig. 1:**
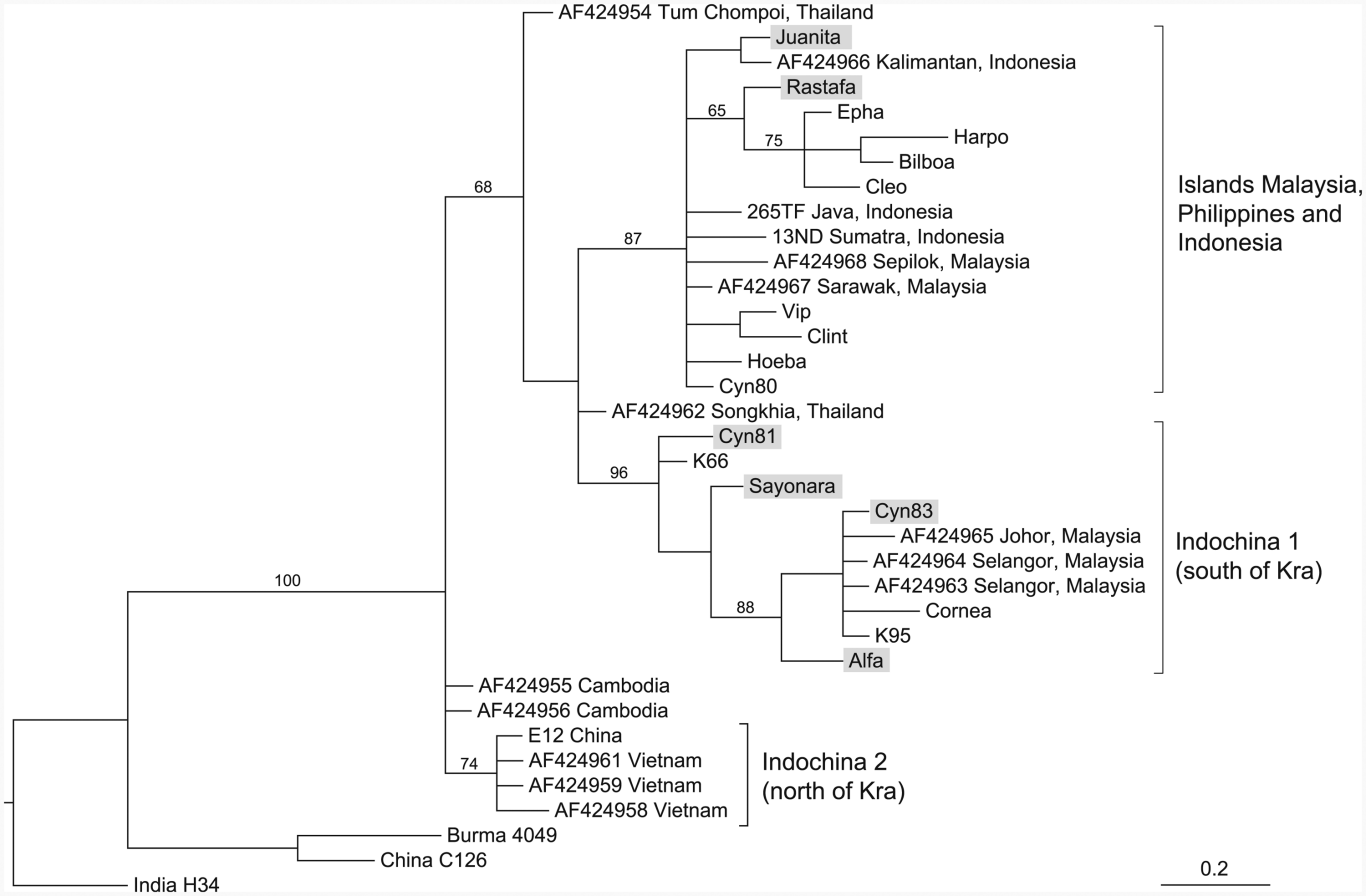
Phylogenetic tree of mtDNA sequences coding for parts of the 12S rRNA of cynomolgus macaques. *Brackets* indicate the different geographic clusters. The tree was rooted with selected rhesus macaque sequences from India, China, and Burma. mtDNA sequences without accession numbers have been published recently (Doxiadis et al. 2006a) or have now been submitted to EMBL/GeneBank database. Juanita, Rastafa, Alfa, and Sayonara are representatives of the four Cynomolgus families, and Cyn81 and Cyn83 are the two unrelated animals analyzed in this study (*shadowed*)

Sequencing of the 3′ part of the 12S rRNA has been performed of cynomolgus monkeys of an outbred breeding colony, and all different mtDNA sequences detected were subjected to phylogenetic analysis in comparison to published mtDNA sequences of established geographic origin. The resulting tree reveals a differential clustering of mtDNA sequences reflecting the origin of the monkeys (Fig. 1). A main branching point can be observed separating mtDNA segments of cynomolgus macaques from Indochina, north of the isthmus of Kra including China and Vietnam on the one hand and Malaysia and the Indonesian/Malaysian islands on the other hand. These data confirm earlier observations that monkeys from Indochina and Malaysia/Indonesia are phylogenetically distinguishable by mtDNA analysis (Smith et al. 2007). Although the two samples from Thailand that are not supported by bootstrap values do not cluster together with the mtDNA sequences of animals from north of Kra, the data indicate that the families of Juanita and Rastafa originated from the Malaysian/Indonesian islands, whereas the families of Alfa and Sayonara, and the two unrelated monkeys, Cyn81 and Cyn83, have their roots in the Malaysian peninsula, south of the isthmus of Kra (Fig. 1).

### *Mafa*-*DRB* haplotype definition by DRB-STR genotyping

**Fig. 2:**
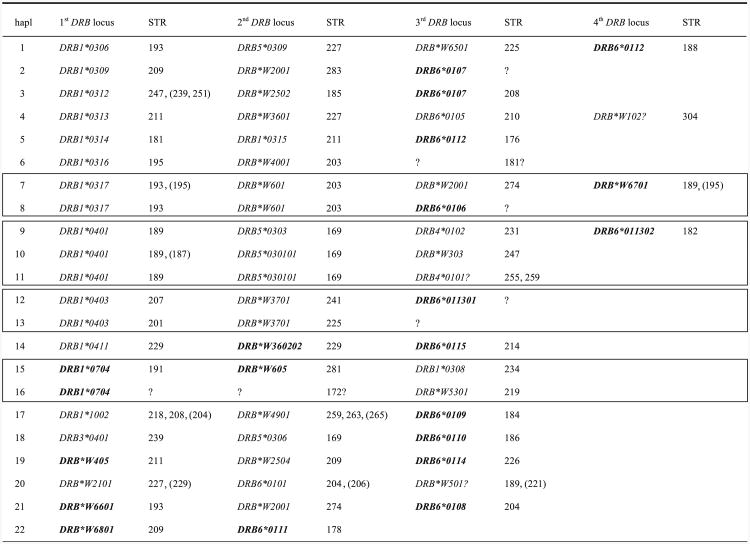
*Mafa-DRB* haplotypes defined by exon 2 sequencing and DRB-STR genotyping. Data *in parentheses* are STR length observed in some (one or two) animals. *Question marks* indicate STRs not detected or rarely detected but the presence of a gene is ascertained by sequencing. 172? and 181? indicate that these STRs were detected but not confirmed by sequencing. DRB4*0101? and DRB*W501? are detected on cDNA, but not on gDNA most likely due to primer inconsistency. *Mafa-DRB* alleles which are represented in *bold* have not been reported earlier

The animals studied, comprising four pedigreed families, are part of a self-sustaining breeding colony. The members of the Alfa family had been characterized previously for their *Mafa-A* alleles, and haplotypes were determined by segregation analyses (Otting et al. 2007). In addition to segregation analyses, in this study, *DRB* haplotypes were defined by the combination of certain DRB-STR alleles and exon 2 sequences in at least two different PCR reactions. As shown for humans and rhesus macaques, all intact *Mafa-DRB* genes appear to possess the D6S2878 microsatellite. Therefore, the panel of 71 animals was analyzed in depth. The DNA segment covering the microsatellite was amplified by means of a single primer set that had previously been developed for studying rhesus monkeys (Doxiadis et al. 2007). Most of the relevant DRB-STR segments, covering most of the known lineages and loci, were successfully amplified. Amplification failures have only been observed for three *DRB6* alleles, of which the corresponding exon 2 sequences were also difficult to detect. This failure was most probably a result of primer inconsistencies, possibly due to the instability of this particular pseudogene. However, the overall data illustrate that an analysis of each monkey sample resulted in the detection of four to eight amplicons, the lengths of which are highly variable and range from 169 to 304 bp. Subsequent sequencing of the relevant DNA segments was conducted to link each DRB-STR allele unequivocally to a particular *DRB* gene/allele. In total, 60 different *Mafa-DRB* alleles could be defined, 18 of which had not yet been described (Fig. 2). With only two exceptions, each *Mafa-DRB* allele could be linked to a certain DRB-STR. On average, most of the STR linked to a particular allele appeared to be conservative in composition and length, and, above all, segregated in a Mendelian manner. Various STR markers seem to be highly predictive for the presence of a particular *Mafa-DRB* allele. For example, no length variability is observed for the DRB*W2502- and DRB5*030101-associated STR markers in the frequently observed haplotypes 3 and 10, respectively (Fig. 2). Some markers, however, like the DRB1*0312-linked STR of haplotype 3, for example, display slight length differences. These are not experimental artifacts, as these structures originate on different founder haplotypes, as was proven by plotting their segregation in cynomolgus families (Fig. 2).

Based on the unique combinations of certain DRB-STR/exon 2 alleles and subsequent segregation analyses, 22 different *Mafa-DRB* haplotypes characterized by the presence of two to four distinct *DRB* genes were defined (Fig. 2). As in humans and rhesus macaques, the combination of different DRB-STR markers appears to be unique for a given haplotype. *DRB* haplotypes of all but two monkeys could be determined. The two exceptions represent animals that are unrelated to the rest of the panel. Thus, this technique can be used to study other Old World monkeys that have complex *DRB* regions such as mandrills (Abbott et al. 2006) and baboons (Huchard et al. 2006); this approach is especially powerful if family samples are available.

### *Mamu*- versus *Mafa*-*DRB*: allelic variation, diversity, and region configurations/haplotypes

The present study shows that numbers of *DRB* alleles, lineages, and region configurations observed in the cynomolgus macaques studied are comparable to the rhesus monkey system that was described earlier (Doxiadis et al. 2007) (Fig. 2). One particular *Mafa-DRB* region configuration has also been observed in Chinese rhesus macaques, indicating that this configuration may predate speciation, and thus represent an evolutionarily old entity (Fig. 2, haplotype 3). However, introgression of rhesus macaques into cynomolgus macaques near the isthmus of Kra has been described, and therefore the possibility of a mixture of both species cannot be excluded (Tosi et al. 2002). Both panels comprised animals of pedigreed macaque families. However, in the former study, twice the number of rhesus macaques was analyzed. Furthermore, the *Mamu-DRB* region was mainly studied in monkeys of Indian origin, which, however, made up about 60% of the region configurations determined (Doxiadis et al. 2007). Based on these comparisons, it can be concluded that cynomolgus macaques possess more allelic variation as well as a higher level of *DRB* region configuration polymorphism in comparison to Indian rhesus macaques. These results are in agreement with mtDNA analyses, which suggest that Indian rhesus macaques were reproductively isolated from Chinese monkeys during much of the Pleistocene and may have experienced a severe genetic bottleneck (Smith and McDonough 2005; Smith et al. 2007). However, Indian and Chinese rhesus macaques show higher levels of gene copy number variation, as two to six DRB loci are present per haplotype (Doxiadis et al. 2007). In contrast, in cynomolgus macaques, most of the region configurations are composed of three *DRB* loci, and more than four loci per haplotype have not been observed (Fig. 2). The composition of the *DRB* region configurations of the two macaque species also shows marked differences. Some *DRB* region configurations of the rhesus monkey display limited levels of allelic polymorphism, whereas no allelic variation can be observed for the *Mafa-DRB* region configurations (Khazand et al. 1999; Doxiadis et al. 2000; Doxiadis et al. 2007). It is noteworthy that the allelic polymorphism of rhesus macaques has only been observed between either monkeys of different origin or between animals of Indian origin. Since the Indian rhesus macaques seem to have run through a bottleneck, it may be speculated that these monkeys built up their genetic repertoire afterwards by the creation of allelic variation instead of haplotype diversity (Hernandez et al. 2007; Smith et al. 2007).

Several *Mafa-DRB* haplotypes share alleles/loci that are identical for their exon 2 sequences, whereas the other DRB genes on the chromosome are different (Fig. 2, bordered). An example is provided by haplotypes 9, 10, and 11, which all share the Mafa-DRB1*0401 allele together with a DRB5*03 lineage member. Furthermore, haplotypes 9 and 11 encode a DRB4*01 allele, whereas haplotype 10 possesses a DRB*W3 allele instead. Haplotype 9, however, has an additional *DRB6* locus that is absent on haplotype 11. This sharing of haplotype segments suggests that, in cynomolgus monkeys, even more frequently than in rhesus macaques, new region configurations are generated by recombinationlike events.

### Evolutionary history of the DRB-STR in macaques

Comparison of *DRB* sequences obtained from different species indicates that the ancestral structure of the DRB-STR most likely must have been a (GT)x(GA)y dinucleotide repeat (Riess et al. 1990; Bergstrom et al. 1999; Kriener et al. 2000; Doxiadis et al. 2007). Nearly all *HLA*-and *Mamu-DRB* gene-associated microsatellites thus far encountered are constructed of four sections, namely (GT)x (GA)z-mix(GA)y(GC)n, exhibiting differential evolutionary stabilities (Doxiadis et al. 2007). The 5′(GT) repeat represents habitually the longest and uninterrupted part, and evolves most rapidly. The second (GA)z part is often shorter and interrupted by other dinucleotides; its composition correlates well with different *DRB* loci/lineages. In the case of *HLA-DRB1*, the length of the third (GA)y segment often correlates with lineages as well, whereas in rhesus macaques and for other *HLA-DRB* loci, such a correlation is less prominent. The 3′ end of the repeat is formed by a short (GC)n part.

**Fig. 3:**
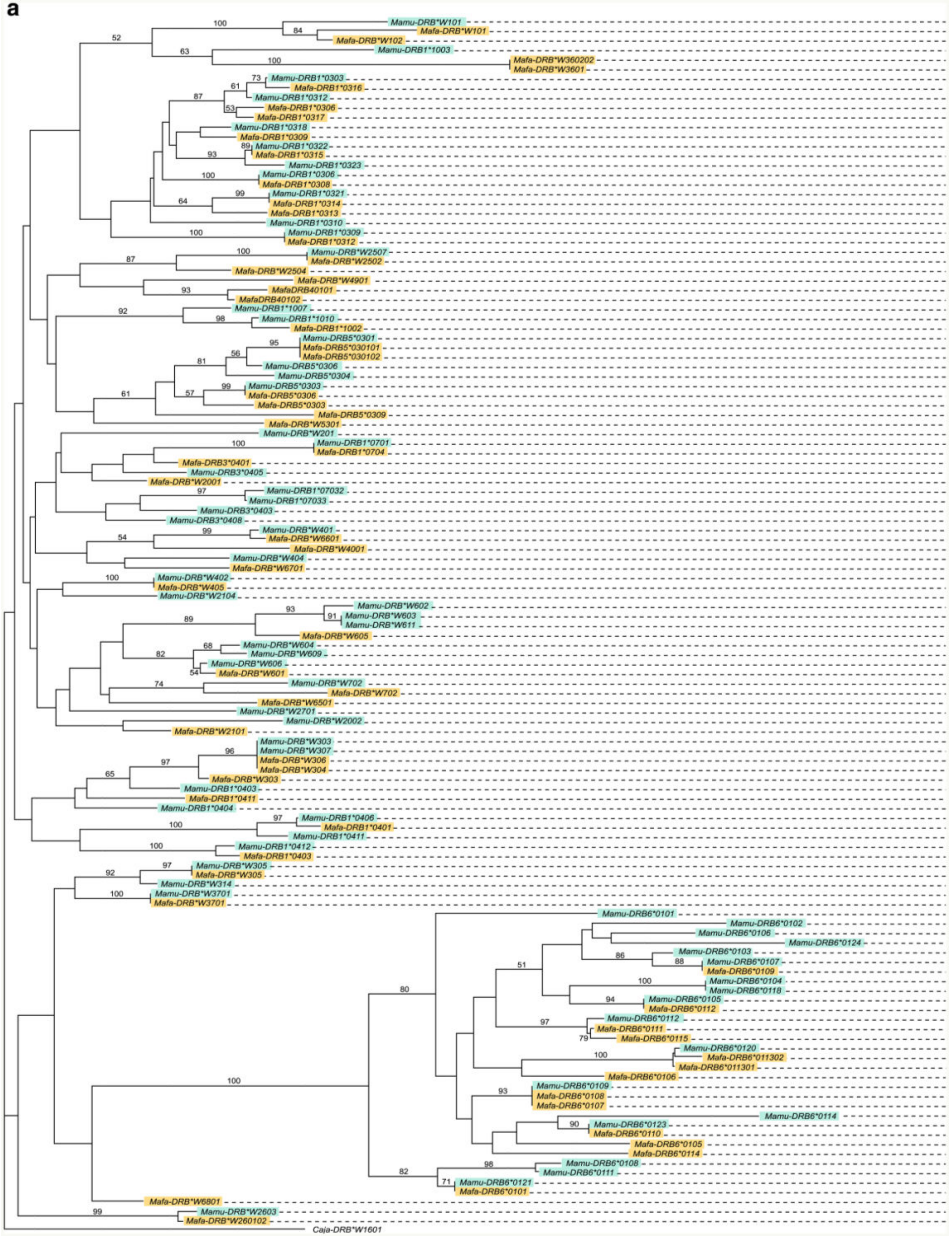

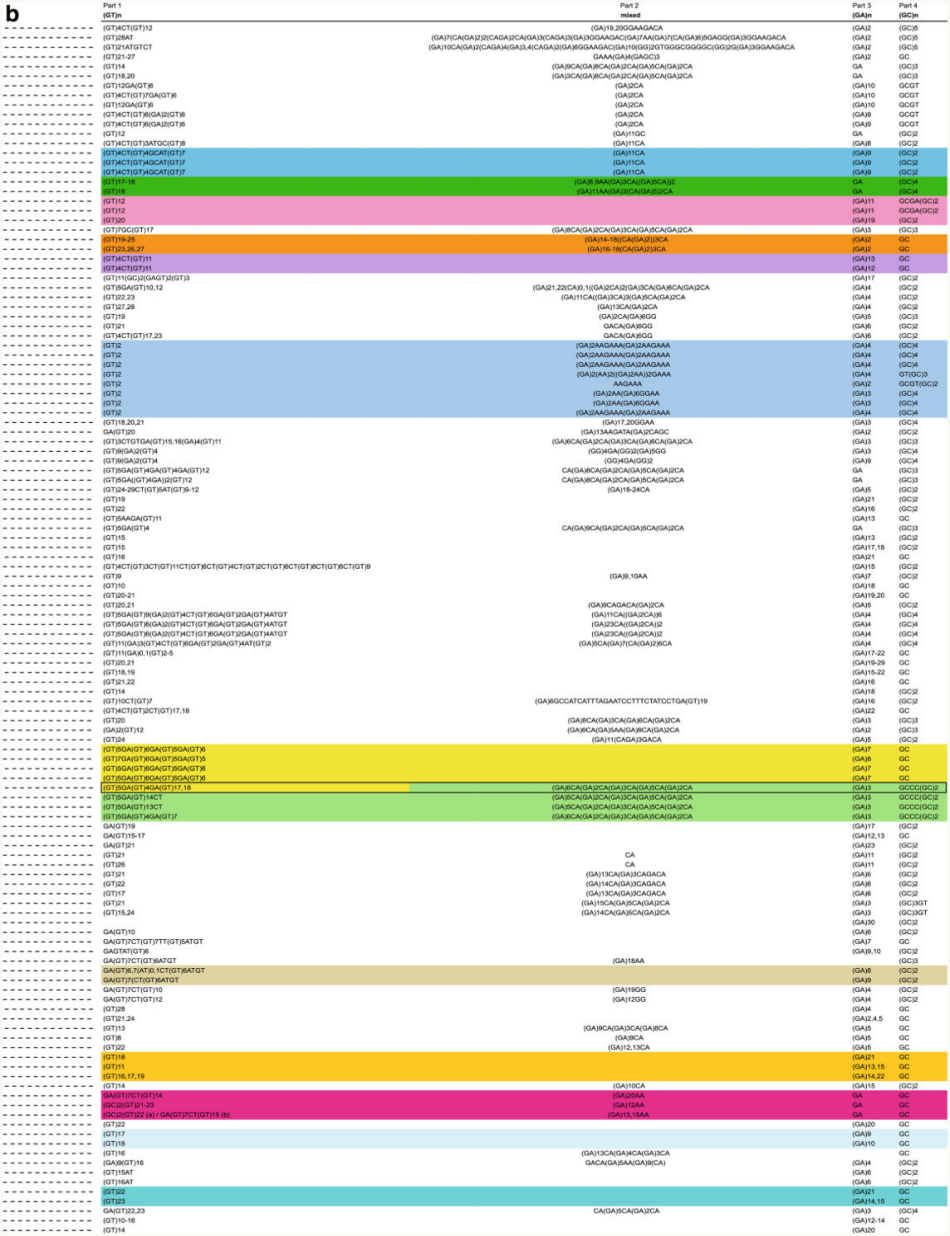
**a** Phylogenetic tree of *DRB* exon 2 sequences of rhesus and cynomolgus macaques. *DRB* alleles of cynomolgus and rhesus macaques are depicted in *yellow and blue boxes*, respectively. **b** Composition of the DRB-STR microsatellite The order of the sequences follows the order of the exon 2 sequences as they cluster in the phylogenetic tree (**a**). Clustering of alleles shared between both macaque species is illustrated by color codes; the bordered sequence (*yellow* and *green*) highlights a crossing-over event. (a) (GT)n part of Mafa-DRB6*0107 belonging to haplotype 2, (b) (GT)n part of Mafa-DRB6*0107 belonging to haplotype 3

The *Mafa-DRB* sequences described in this study were analyzed together with the *Mamu-DRB* alleles published previously (Doxiadis et al. 2007) (Fig. 3a), and the corresponding microsatellite sequences have been superimposed (Fig. 3b). The high number of branches as well as the complex microsatellite patterns correlates with the high number of *DRB* region configurations/haplotypes observed in rhesus as well as in cynomolgus macaques. As one would expect, the *Mamu*- and *Mafa-DRB* sequences intermingle in the phylogenetic tree, and alleles of identical loci/lineages of both species cluster together. *Mamu/Mafa-DRB6* alleles form a distinct clade in the tree, which is in agreement with the fact that this locus is an old entity predating the divergence of Old World monkeys and humans and great apes (Fig. 3a). Furthermore, the composition of the microsatellite is alike in both macaque species, reflecting their common ancestry (Fig. 3b). Such sharing of similar sequence motifs has been highlighted by similar color codes (Fig. 3b). An example is given for the *Mamu/Mafa-DRB* alleles of the DRB*W3 lineage, which appear to be biphyletic, since they cluster on two branches in the tree (Fig. 3a and b). The composition of the adjacent DRB-STR matches the phylogeny (Fig. 3b, yellow). One of the DRB*W3 alleles of the second group, Mafa-DRB*W303, clusters slightly apart, next to DRB1*04 lineage members (Fig. 3b, green). As can be seen, its STR composition appears to be a mixture of the two lineages. The (GT)x part resembles the first part of the DRB*W3 members (Fig. 2b, yellow), the following parts of the STR are the same as in the DRB1*04 lineage (Fig. 3b, green). Thus, the genetic makeup of this DRB-STR suggests that it was generated by a crossing-over event between two different *DRB* lineages. This type of result underscores the above-described notion (Fig. 2, haplotypes 9, 10, and 11) that the generation—also called the birth and death process—of *DRB* genes and haplotypes is a steadily ongoing process, mostly propelled by recombination-like processes. These data are in concert with recent observations that illustrate that the *DRB* genes themselves, judged from an evolutionary perspective, are relatively young entities (von Salome et al. 2007), but that exon 2 gene segments that encode the peptide binding site represent much older gene segments that are frequently sprinkled over different duplicated DRB genes during primate evolution (Doxiadis et al. 2008b).

### Identical *Mamu* and *Mafa*-*DRB* alleles and their STR compositions

Rhesus and cynomolgus macaques share not only lineages but also a considerable number of identical *Mhc-DRB* alleles (Blancher et al. 2006; Doxiadis et al. 2006a). This high level of sharing of *DRB* sequences is unique. In the present panel, 13 *DRB* exon 2 sequences are encountered that are identical in both species. The microsatellites belonging to the identical pairs of alleles have been compared, and only four sets share the same DRB-STRs (Table 1, nos. 3, 4, 6a/6b, and 7). These microsatellites are characterized by relatively short and/or composed repeats and are considered to be more stable. For instance, Mamu-DRB5*0301 is identical to two *Mafa-DRB5* alleles; one based on the nucleotide and the other on the deduced amino acid sequence. (Table 1, no. 6a/6b). However, most of the identical *Mafa/Mamu-DRB* exon 2 pairs segregate with slightly different DRB-STR lengths, indicating that the STR evolved faster than the adjacent exon (Table 1). For example, Mafa-DRB6*011301, identical to Mamu-DRB6*0120, has an adjacent DRB-STR of 192 and 208 bp length, respectively, with a composition somewhat different in number of (GT)x and (GA)y repeats. Another example is provided by the DRB-STR linked to the indistinguishable Mamu-DRB1*0309 and Mafa-DRB1*0312 exon 2 sequences. Both are highly variable in length, which is most likely due to their long and uninterrupted (GT) and (GA) repeats of the first and second part of the STR. Furthermore, the two alleles are part of the *DRB* haplotype, which is shared between both macaque species. Therefore, this fast and accurate DRB-STR genotyping method is not only valuable for various research lines but also appears to be helpful in discriminating between different macaque species. As such, this microsatellite will be a useful extension of other markers that were described earlier (Penedo et al. 2005; O'Connor et al. 2007) to type the MHC of macaques.

### Concluding remarks

The *DRB* region of humans as well as macaque species has been subjected to several rounds of duplication and contraction processes caused by a complex series of recombination-like events. Different mechanisms have been proposed for the instability of the macaque *DRB* region. For instance, sense- or antisense integration of intronic endogenous retroviruses may influence the stability or instability of primate *DRB* genes/region configurations (Doxiadis et al. 2008a). Here, a recombination event is reported that maps within a compound microsatellite. It is even possible that the microsatellite itself influences the stability of the *DR* region by promoting recombination-like events. At this stage, it is difficult to determine whether highly related genes located on different region configurations represent separate loci or whether these sequences have an allelic affiliation. Bearing in mind the results described above, this microsatellite will be helpful in sorting out paralogous and orthologous relationships.

## Figures and Tables

**Table 1:** *Mafa* class II alleles detected and their identity to *Mamu* orthologue

	Alleles	STR composition			
1	Mamu-DRB1*0306	(GT)_17–18_	(GA)_8,9_AA(GA)_3_CA((GA)_5_CA)_2_	GA	(GC)_4_
	Mafa-DRB1*0308	(GT)_18_	(GA)_11_AA(GA)_3_(CA(GA)_5_)_2_CA	GA	(GC)_4_
2	Mamu-DRB1*0309	(GT)_19–25_	(GA)_14–18_(CA(GA)_2_)_3_CA	(GA)_2_	GC
	Mafa-DRB1*0312	(GT)_23,26,27_	(GA)_16–18_(CA(GA)_2_)_3_CA	(GA)_2_	GC
3	Mamu-DRB1*0321	(GT)_12_		(GA)_11_	GCGA(GC)_2_
	Mafa-DRB1*0314	(GT)_12_		(GA)_11_	GCGA(GC)_2_
4	Mamu-DRB1*0322	(GT)_4_CT(GT)_4_GCAT(GT)_7_	(GA)_11_CA	(GA)_9_	(GC)_2_
	Mafa-DRB1*0315	(GT)_4_CT(GT)_4_GCAT(GT)_7_	(GA)_11_CA	(GA)_9_	(GC)_2_
5	Mamu-DRB1*0303^a^	(GT)_12_GA(GT)_6_	(GA)_2_CA	(GA)_10_	GCGT
	Mafa-DRB1*0316^a^	(GT)_4_CT(GT)_7_GA(GT)_6_	(GA)_2_CA	(GA)_10_	GCGT
6a	Mamu-DRB5*0301	(GT)_2_	(GA)_2_AAGAAA(GA)_2_AAGAAA	(GA)_4_	(GC)_4_
	Mafa-DRB5*030101	(GT)_2_	(GA)_2_AAGAAA(GA)_2_AAGAAA	(GA)_4_	(GC)_4_
6b	Mamu-DRB5*0301^a^	(GT)_2_	(GA)_2_AAGAAA(GA)_2_AAGAAA	(GA)_4_	(GC)_4_
	Mafa-DRB5*030102^a^	(GT)_2_	(GA)_2_AAGAAA(GA)_2_AAGAAA	(GA)_4_	(GC)_4_
7	Mamu-DRB5*0303	(GT)_2_	(GA)_2_AA(GA)_6_GGAA	(GA)_3_	(GC)_4_
	Mafa-DRB5*0306	(GT)_2_	(GA)_2_AA(GA)_6_GGAA	(GA)_3_	(GC)_4_
8	Mamu-DRB6*0121	(GT)_22_		(GA)_21_	GC
	Mafa-DRB6*0101	(GT)_23_		(GA)_14,15_	GC
9	Mamu-DRB6*0109	GA(GT)_7_CT(GT)_14_	(GA)_20_AA	GA	GC
	Mafa-DRB6*0107	GA(GT)_7_CT(GT)_15_	(GA)_13,15_AA	GA	GC
10	Mamu-DRB6*0107	GA(GT)_6,7_(AT)_0,1_CT(GT)_6_ATGT		(GA)_8_	(GC)_2_
	Mafa-DRB6*0109	GA(GT)_7_(CT(GT)_6_ATGT		(GA)_9_	(GC)_2_
11	Mamu-DRB6*0123	(GT)_17_		(GA)_9_	GC
	Mafa-DRB6*0110	(GT)_18_		(GA)_10_	GC
12	Mamu-DRB6*0120	(GT)_18_		(GA)_21_	GC
	Mafa-DRB6*011301	(GT)_16,17,19_		(GA)_14,22_	GC
13	Mamu-DRB*W2507	(GT)_4_CT(GT)_11_		(GA)_13_	GC
	Mafa-DRB*W2502	(GT)_4_CT(GT)_11_		(GA)_12_	GC

a Alleles are not identical on nucleotide level but on amino acid level they are the same
